# Supplementary material for: Identification of photoperiod-induced specific miRNAs in the adrenal glands of Sunite sheep (Ovis aries)
Source: Front Vet Sci. 2022 Jul 22;9:888207. doi: 10.3389/fvets.2022.888207 (PMC9354845; doi:10.3389/fvets.2022.888207)
Supplement: Supplementary file 1 [file Data_Sheet_1.ZIP › Supplementary file/miRDeep2 file.zip!/miRDeep2 file/Novel_Structure/SPLP42A2/Novel_200.pdf]

| | | |
|---|---|---|
| Provisional ID | : | NC_019459.2_4558 |
| Score total | : | 2.6 |
| Score for star read(s) | : | -1.3 |
| Score for read counts | : | 0 |
| Score for mfe | : | 2.3 |
| Score for randfold | : | 1.6 |
| Score for cons. seed | : | |
| Total read count | : | 18 |
| Mature read count | : | 18 |
| Loop read count | : | 0 |
| Star read count | : | 0 |

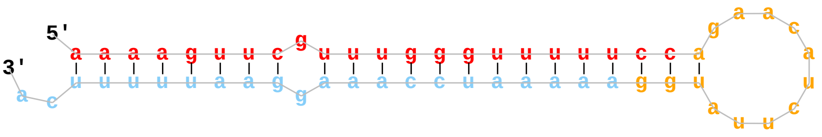


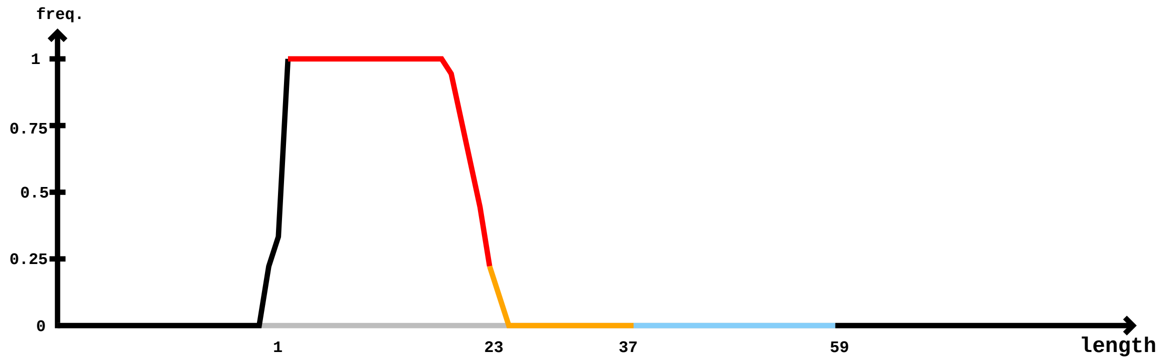


**Mature** **Star**

```
5'- ucaaauaauauuggguuggcugaaaaguucguuuggguuuuuccagaacaucuuauggaaaaauccaaaggaauuuuucagccaacacaauauuaacuuucacagcuuug   -3'    exp
    .....(((((((.(((((((((((((((.(((((((((((((..........))))))))))))).))))))))))))))).))))))).....................   reads      mm         sample
    ....................ugaaaaguucguuuggguuu......................................................................   2          0          seq
    ....................ugaaaaguucguuuggguuuu.....................................................................   2          0          seq
    .....................gaaaaguucguuuggguuuuu....................................................................   2          0          seq
    ......................aaaaguucguuuggguu.......................................................................   1          0          seq
    ......................aaaaguucguuuggguuu......................................................................   1          0          seq
    ......................aaaaguucguuuggguuuG.....................................................................   1          1          seq
    ......................aaaGguucguuuggguuuuu....................................................................   1          1          seq
    ......................aaaaguucguuuggguuuuuA...................................................................   2          1          seq
    ......................aaaaguucgCuuggguuuuuc...................................................................   2          1          seq
    ......................aaaaguucguuuggguuuuucG..................................................................   3          1          seq
    ......................aaaaguucguuuggguuuuucAa.................................................................   1          1          seq
```
